# Report of Deaths in the City of Buffalo, for the Month of July, 1864

**Published:** 1864-09

**Authors:** Sandford Eastman

**Affiliations:** Health Physician


*Report of Deaths in the City of Buffalo, for the month of July,* 1864.

SEX.—Males, 100; Females, 60.

CAUSES OF DEATH.—Accident, 7; Accident, by Drowning, 6; Apoplexy, Cerebral, 1; Brain, congestion of, 1; Bronchitis, 4; Cholera Infantum, 20; Cholera Morbus, 2; Consumption, 2; Convulsions, 8; Croup, 1; Coxalgia, 1; Debility, 5; Diarrhœa, 16; Disease of the Brain, 1; Disease of the Heart, 1; Diptheria, 2; Dropsy, General, 2; Dysentery, 4; Epilepsy. 2; Erysipelas, 2; Fever. Puerperal, 1; Fever, Pueprperal Convulsive, 1; Fever, Scarlet, 2. Fever, Typhoid, 3; Gangrene, (Hosp.) 1; Gunshot Wound, 3; Hæmorrhage, from Lungs, 1; Inflammation of Bowels, 2; Inflammation of Brain, 5; Inflammation of Brain and Meninges, 6; Inflammation of Larynx, 1; Inflammation of Liver, 1; Inflammation of Lungs, 8; Inflammation of Peritoueum, 1; Marasmus, 4; Measles, 6; Murder, 4; Old Age, 4; Pyæmia, 2; Perforation of Bowels, 1; Scrofula, 1; Small-pox, 1; Stricture of Rectum, 1; Strangulation, 1; Unknown, 3.

Total Deaths from Diseases, 168; Still-born, 1.

LOCALITY.—City at large, 144; Hospital Sisters of Charity, 3; Buffalo General Hospital, 4; Catholic Foundling Asylum, 11; Catholic Deaf and Dumb Asylum, 1; Protestant Orphan Asylum, 1; Erie County Alms House, 4; Soldiers' Rest, 2.

BY WHOM CERTIFIED.—By Regular Physicians at Public Institutions, 25; by Regular Physicians in City at large, 74; by Irregular Practitioners, 31; by Coroner, 15; by Undertakers, 24—Total, 169.

The number of deaths for the first six months of the present year is 109 more than in the corresponding period of last year, and 80 more than the average for five years.

SANDFORD E ASTMAN, M. D. Health Physician.

